# Léiomyosarcome utérin récidivant: à propos d'un cas

**DOI:** 10.11604/pamj.2014.19.74.4565

**Published:** 2014-09-24

**Authors:** Youssef Benabdejlil, Mohammed Elmarjany, Abdellah Babahabib, Mehdi Elhassani, Hafsa Chahdi, Jaouad Kouach, Driss Rahali Moussaoui, Mohammed Dehayni

**Affiliations:** 1 Service Gynécologie-Obstétrique, Hôpital Militaire d'Instruction Mohammed V, Rabat, Maroc; 2 Service de Radiothérapie, Hôpital Militaire d'Instruction Mohammed V, Rabat, Maroc; 3 Service Anatomie-Pathologique, Hôpital Militaire d'instruction Mohamed V, Rabat, Maroc

**Keywords:** Léiomyosarcome, hystérectomie, pronostique, leiomyosarcoma, hysterectomy, prognostic

## Abstract

Les léiomyosarcomes utérins sont des tumeurs malignes, rares, de mauvais pronostic. Nous rapportons un cas de léiomyosarcome diagnostiqué initialement à l'examen anapath d'une pièce d'hystérectomie réalisée pour myome utérin chez une femme de 45 ans. Il s'agit d'une observation particulière. La plupart des auteurs soulignent le mauvais pronostic précoce du léiomyosarcome avec récidive tumorale; Le principal facteur pronostique étant l'activité mitotique. La tumeur de notre patiente a une activité mitotique élevée, elle a récidivé trois fois en cinq ans et opérée à trois reprises.

## Introduction

Les sarcomes utérins font partie des cancers utérins rares et représentent entre 2 et 6% des tumeurs malignes du corps de l'utérus [1, 2]. Ce sont des tumeurs de mauvais pronostic caractérisés par une grande hétérogénéité sur le plan anatomopathologique. S'il est admis que le traitement de référence des sarcomes utérins est chirurgical, la place des traitements adjuvants reste discutée [1, 3, 4]. Les auteurs rapportent un cas de léiomyosarcome récidivant en précisant ses caractéristiques cliniques, radiologiques, histopathologiques et discutent les difficultés diagnostiques et thérapeutiques.

## Patient et observation

**Figure 1:**
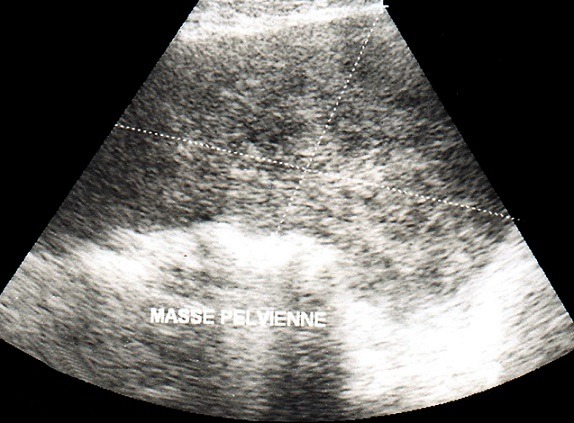
Échographie pelvienne par voie sus pubienne réalisée dans le grand axe de la tumeur et montrant une volumineuse masse d’échostructure hétérogène

**Figure 2:**
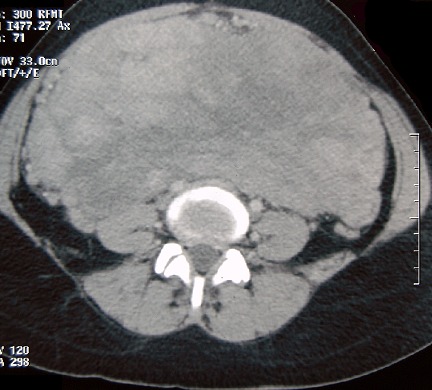
TDM abdomino-pelvienne sur le plan axial: volumineuse masse abdomino-pelvienne de densité hétérogène avec des zones tissulaires rehaussées après injection de produit de contraste iodé avec présence de zones hypodenses non rehaussées correspondants à des remaniements kystiques et nécrotiques

Mme BY âgée de 45 ans, 5^ème^ geste, 5^ème^ pare, sans antécédents pathologies notables, de bas niveau socio-économique, a été admise dans notre service en février 2008 pour douleurs pelviennes chroniques associées à des ménométrorragies compliquées d'un syndrome anémique sévère. L'examen clinique a retrouvé une patiente en mauvais état général, ses conjonctives décolorées, la palpation abdominale a trouvé une masse pelvienne ferme indolore et à l'examen gynécologique le col était macroscopiquement sain, le toucher vaginal a trouvé l'utérus augmenté de taille à mi-chemin ombilic-pubis avec une masse de consistance ferme solidaire de l'utérus bombant dans le cul de sac de douglas. Le reste de l'examen général était sans particularité. L’échographie pelvienne trouvait l'utérus augmenté de taille avec un myome interstitiel faisant 82/58mm. Les annexes étaient sans anomalies. L'hémogramme a objectivé une anémie hypochrome microcytaire à 5,6/dl. Le reste du bilan préopératoire était sans anomalies. La patiente a reçu 3 concentrés globulaire en préopératoire. A l'exploration chirurgicale l'utérus était augmenté de volume siège d'un myome postérieur. L'ovaire gauche et l'annexe droite paraissent sains. Une hystérectomie totale avec annexectomie gauche sont réalisées. Les suites opératoires étaient simples. L’étude histologique était en faveur d'un léiomyosarcome utérin grade II avec salpingite gauche aigue ulcérée et suppurée, l'ovaire gauche était sain. Devant ce résultat, une décision de complément thérapeutique chirurgical plus ou moins radio chimiothérapie est décidé sauf que la patiente est perdue de vue. Quatre années après, elle consulte pour augmentation du volume de l'abdomen associé à une pesanteur pelvienne et amaigrissement non chiffré, sans signes urinaires ou digestifs. L'examen général retrouvait une patiente en assez bon état général, apyrétique. L'examen de l'abdomen retrouvait à l'inspection, une volumineuse voussure abdomino-pelvienne arrivant jusqu’à l’épigastre. A la palpation, cette masse est mal limitée, mobile, indolore faisait 30/20 cm et de consistance ferme. A l'examen gynécologique, la tranche vaginale était saine et souple. Le Cul de sac de Douglas était comblé par la masse abdominale. L’échographie abdomino-pelvienne objectivait une masse volumineuse d'aspect hétérogène faisant 25/18 cm (Figure 1). Le scanner abdomino-pelvien, retrouvait une masse abdomino-pelvienne hétérogène avec des zones charnues rehaussées après injection du produit de contraste, délimitant des zones kystiques de nécrose (Figure 2). Cette masse mesurait 26/21/10 cm et refoulait les structures digestives en avant et latéralement arrivant en haut au contact du colon transverse et de la paroi abdominale antérieur en avant. On note également la présence d'une urétéro-hydronéphrose bilatérale plus marquée à droite. Le scanner thoracique était normal.

**Figure 3:**
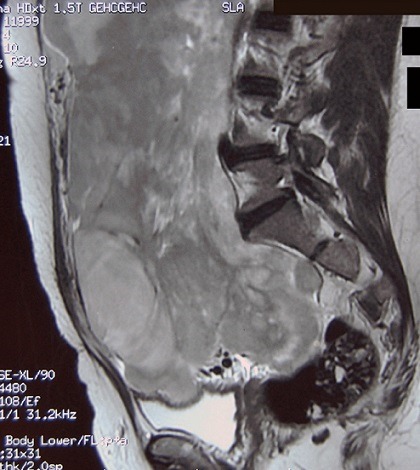
IRM abdomino-pelvienne: Séquence T2 en écho de spin sagittale montrant une volumineuse masse en iso-hyper signal T2, bien délimitée, très hétérogène, renfermant des plages de signal kystique en hypersignal T2 correspondants à des zones de nécrose. Cette volumineuse masse remplie la quasi-totalité de la cavité abdominale inférieure et réalise un effet de masse sur les organes de voisinage

**Figure 4:**
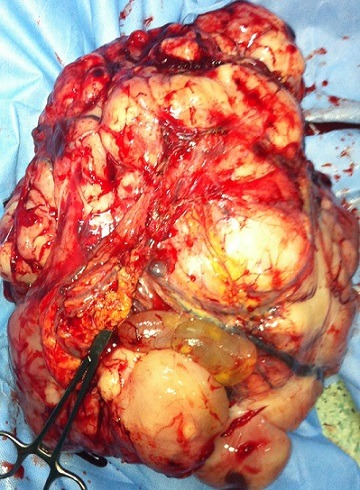
Aspect macroscopique de la tumeur en per opératoire et après sa resection

**Figure 5:**
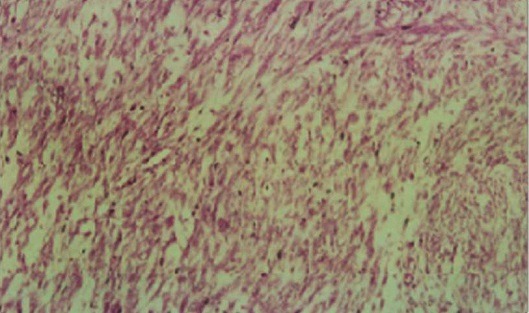
Leiomyosarcome faible grossissement

**Figure 6:**
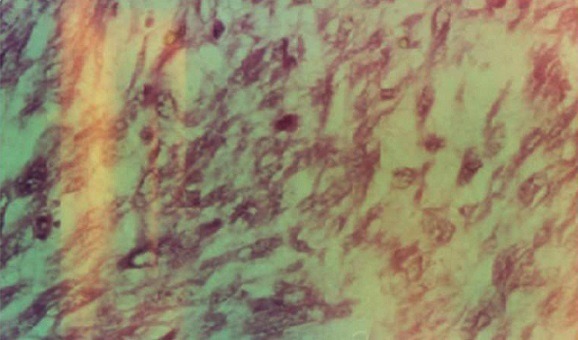
Leiomyosarcome HES x 400: prolifération de cellules fusiformes ou étoilées avec irrégularité des noyaux et présence de mitoses

L'IRM pelvienne a objectivé un volumineux processus abdomino-pelvien, de signal tissulaire hétérogène, renfermant des zones de nécrose, mesurant 21/11/25 cm (Figure 3). Il refoule les anses digestives latéralement, occupe la loge utérine en bas et arrive au contact de la face supérieure de la vessie et antérieure du rectum avec persistance d'un liseré graisseux de séparation. Ce processus refoule également les divisions vasculaires iliaques en arrière et en dehors. De plus, on note la présence d'une lame d’épanchement liquidienne intra péritonéal. Le diagnostic de récidive du léiomyosarcome était retenu et la patiente a bénéficié d'une deuxième laparotomie exploratrice réalisée le 31/05/2012 associé à une montée d'une sonde JJ en per opératoire. A l'exploration, on notait la présence d'une ascite de moyenne abondance à liquide jaune claire, un nodule dans le douglas qui était réséqué et une volumineuse masse abdomino-pelvienne faisant 40/30 cm, irrégulière adhérente à l’épiploon siège d'une néo vascularisation anarchique (Figure 4), le tube digestif était libre. La résection en totalité de la tumeur fut réalisée avec annexectomie droite et appendicectomie. L’étude histologique a objectivé une prolifération tumorale mésenchymateuse maligne, elle est faite de cellules fusiformes, aux noyaux en bout de cigare hyper chromatique avec un index mitotique estimé à 24 mitoses\10 champ au fort grossissement. Ces cellules sont disposées en faisceaux long entrecroisés à angle aigue. Sur deux lames, on retrouve des plages de nécrose de coagulation (Figure 5, Figure 6). Ces éléments sont en faveur d'une récidive d'un léiomyosarcome de grade II, l'annexe droite était saine et l'appendice sans anomalie. Le nodule du douglas était le siège de remaniements fibreux et la cytologie péritonéale était normale. Les suites post opératoires étaient simples.

**Figure 7:**
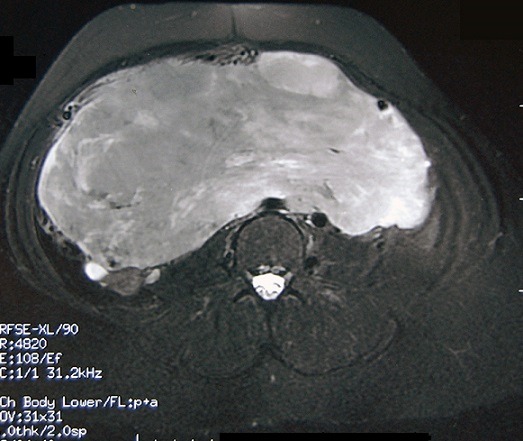
TDM abdominale après injection de produit de contraste iodé en plan axial: Masse abdominale de densité hétérogène, bien délimitée, contenant des zones charnues rehaussées et des zones kystiques de nécrose

La patiente était de nouveau perdue de vue depuis sa sortie. Quatre mois après son intervention, elle consultait pour réapparition d'une masse abdomino-pelvienne perçue depuis une semaine qui avait augmenté progressivement de volume. L'examen de l'abdomen a retrouvé une masse faisant à peu près 10 cm, mobile, de consistance ferme et indolore. L’échographie abdomino-pelvienne a objectivé une masse tissulaire hétérogène mesurant 10 cm de grand axe. Le scanner thoraco-abdomino-pelvien a objectivé une masse abdominale hétérogène bilobée avec des zones charnues rehaussées après injection du produit de contraste, délimitant des zones kystiques de nécrose, mesurant 72/86/14 cm de hauteur. Cette masse refoule les structures digestives en avant et latéralement. Elle arrive en haut au contact du colon transverse et en avant, elle est en contact de la paroi abdominale antérieure. De plus, on note la présence d'une deuxième masse tissulaire pelvienne de 4/10/6 cm de hauteur avec présence d'une lame d’épanchement dans le douglas (Figure 7). Une troisième laparotomie exploratrice est réalisée le 11/10/12. A l'exploration on a noté la présence de multiples nodules tumoraux localisés au niveau de la paroi vésicale, de la paroi rectale, du péritoine pariétal ainsi que du méso sigmoïde. De plus, il ya présence de deux masses niveau du méso transverse faisant respectivement 10 et 6 cm. On a procédé à une exérèse des différents nodules ainsi que des deux masses au niveau du méso transverse puis on a réséqué la partie restante de l’épiploon. Les suites opératoires étaient simples. L’étude histologique a confirmé la récidive sous forme d'une sarcomatose péritonéale. La patiente a bénéficié dans un premier temps d'une première ligne de chimiothérapie à base d'une association de doxorubicine et d'ifosfamide. Après 3 cures, l’évaluation a montré une progression aussi bien clinique que radiologique avec apparition de nouvelles lésions péritonéales et hépatiques. Elle a bénéficié ensuite d'une deuxième ligne de chimiothérapie, à base d'une association de gemcitabine et docetaxel. La patiente est décédée après la fin de la 3ème cure de cette deuxième ligne de chimiothérapie.

## Discussion

Le léiomyosarcome utérin est une tumeur maligne, rare, de nature conjonctive, développée aux dépens des éléments mésenchymateux du myomètre [5]. La fréquence relative est de 1,3% de tous les cancers utérins [6] et correspond à 40 à 50% des sarcomes de l'utérus. Enfin on observe 2 léiomyosarcomes pour 1 000 fibromes utérins [6, 7]. Ils sont caractérisés par une grande hétérogénéité sur le plan anatomopathologique. Ce sont des tumeurs de mauvais pronostic puisque la survie à cinq ans est environ 30% [1–8]. Leur diagnostic doit être précoce, car la survie des patientes est corrélée au stade tumoral [1]. L’âge moyen de survenue varie de 45 à 55 ans [9]. La maladie survient le plus souvent chez des patientes ménopausées depuis 6-7 ans. Notre patiente est âgée de 45 ans, elle est en périménopause. La clinique reste peu spécifique. Les trois signes les plus fréquents que l'on retrouve dans notre observation comme dans la littérature sont: les hémorragies génitales, les douleurs pelviennes et la masse pelvienne [5, 7, 9]. Le diagnostic préopératoire n'est que rarement fait, le léiomyosarcome se présente le plus souvent sous la forme d'un myome banale ou en nécrobiose. Le diagnostic est le plus souvent fait sur une pièce d'hystérectomie réalisée pour fibrome utérin. C'est le cas de notre observation. Classiquement, l'augmentation rapide du volume d'un léiomyome et son ramollissement doivent faire suspecter le diagnostic [6]. L'augmentation rapide d'un léiomyome ne se révèle être un léiomyosarcome que dans 0,27% des cas. L’échographie est peu spécifique [6]; le léiomyosarcome est le plus souvent limité à une seule masse, il n'y a pas de localisation préférentielle et la base est large ou pédiculée. Une augmentation rapide de la taille du fibrome et une image de myome en nécrobiose peuvent être trompeuses. Plusieurs études ont analysé l'apport de l’échographie et du doppler couleur, n'ont pas trouvé de caractères permettant de différencier un fibrome et un sarcome utérin. D'autres auteurs ont comparé les index et le doppler couleur n'ont pas trouvé de différences morphologiques entre les fibromes, les leiomyosarcomes et les carcinosarcomes ou de différences concernant les index doppler entre fibromes et leiomyosarcomes. L'hystérosalpingographie qui est réalisée peut montrer une cavité utérine augmentée de volume, présentant une lacune polycyclique à contours nets, creusée d’échancrures intralésionnelles. Au scanner [10], les lésions se présentent sous forme de larges zones de nécrose ou de transformation kystique, bien que non spécifiques, doivent faire évoquer le diagnostic. L'examen tomodensitométrique permet aussi de rechercher des métastases au niveau du poumon, du mésentère, de l’épiploon, des ganglions rétropéritonéaux et de la rate. Ces lésions métastatiques présentent souvent un centre nécrosé. A l'IRM, les lésions présentent un signal hétérogène en pondération T2 avec des plages en hypersignal. En pondération T1, elles sont en iso- ou en hyposignal comparativement au signal du myomètre. Elles prennent le contraste de manière intense au temps artériel de l'injection; cette prise de contraste est souvent hétérogène. À un temps plus tardif, il est possible d’évaluer s'il existe une nécrose tumorale.[10]

Le diagnostic différentiel est représenté par les fibromes qui sont en hyposignal pondérées T2 et homogènes après injection; la cinétique de la prise de contraste des fibromes est superposable à celle du myomètre. Le problème est celui des fibromes compliqués: les fibromes oedémateux ou en dégénérescence hyaline peuvent présenter un hypersignal en T2; les fibromes en nécrobiose prennent le contraste de manière hétérogène. En revanche, en cas de lésion histologiquement prouvée, l'IRM permet de réaliser au mieux le bilan d'extension locale et locorégionale. Sur le plan anatomopathologique, l'aspect évocateur d'un léiomyosarcome est la consistance molle, friable, la couleur blanche, la taille volontiers volumineuse (en moyenne 10 cm de diamètre), siège de remaniements nécrotiques et hémorragiques avec parfois envahissement franc du myomètre et de la séreuse péritonéale. Les critères histologiques retenus par la plupart des auteurs [5–7] pour le diagnostic de léiomyosarcome sont ceux de Hendrickson et Kempson, repris par Zaloudek et Norris. Le diagnostic est posé lorsqu'il y a plus de 10 mitoses par champ à l'objectif 10 ou s'il y a un nombre de mitoses compris entre 5 et 9 par champ à l'objectif 10 associé à des atypies cellulaires ou des métastases. Silverberg a institué un système de gradation histologique allant des tumeurs bien différenciées aux tumeurs indifférenciées, mais cette classification n'a qu'un intérêt pronostique limité. Les immunomarquages à l'anti-desmine, à l'anti-vimentine, à l'anti-1-anti-trypsine sont utiles pour confirmer le caractère musculaire lisse de la prolifération. L'extension [11] se fait par contiguïté vers le vagin, le pelvis et l'abdomen. Les métastases touchent par ordre de fréquence le poumon, le foie, les os et le cerveau. L'extension lymphatique [11] se fait vers les ganglions pelviens, para-aortiques, mésentériques, médiastinaux, hiliaires et supra-claviculaires. Le facteur pronostique dominant est l'activité mitotique de la tumeur [5, 12], le pronostic est d'autant plus sombre que l'activité mitotique est élevée. Le stade de la maladie influence nettement l’évolution du cancer [12]. L'absence de nécrose et d'une hyalinisation péritumorale sont des facteurs de bon pronostic. Le jeune âge ainsi que la pré-ménopause sont des facteurs de bon pronostic [5, 7, 12].

Tous stades confondus, la survie à 5 ans est estimée entre 25 et 40% [13]. La présence de métastases influence nettement la survie. En effet, la moyenne de survie est de 19 mois lorsqu'il existe une métastase pulmonaire et de 12 mois lorsque plusieurs métastases s'associent. Le décès survient en moyenne 8 mois après l'apparition de la première récidive. Le taux des récidives des léiomyosarcomes varie de 35 à 70% selon les auteurs, touchant le plus souvent le pelvis. Elles surviennent le plus souvent dans les deux ans suivant le diagnostic. Le traitement est essentiellement chirurgical et doit être complet d'emblée [9, 12]. Le premier temps de l'intervention consiste en une cytologie péritonéale et une exploration de l'abdomen. La plupart des auteurs réalisent une hystérectomie non conservatrice, bien que certains aient montré que la conservation des ovaires ne modifie pas la survie. Les gestes complémentaires sont fonction de l'exploration: exérèse viscérale selon l'extension et curage pelvien si des adénopathies sont palpées. La radiothérapie diminue l'incidence des récidives pelviennes mais n'améliore pas la survie globale [9, 13, 14]: sa place doit être discutée au cas par cas.

La chimiothérapie peut être proposée avant chirurgie si la tumeur est jugée non résécable d'emblée ou après chirurgie si la résection tumorale n’était pas optimale ou enfin en cas de métastases à distance. Les principaux protocoles contiennent du cis-platine, de l'adriamycine et de l'ifosfamide et dernièrement le gemcitabine et le docetaxel [13]. L'efficacité de la chimiothérapie dans ce genre de cancers n'est pas encore spectaculaire mais de nouvelles molécules notamment en thérapie ciblée sont en cours d'investigation. Notre patiente a fait une poursuite évolutive sous le protocole associant doxorubicine et ifosfamide et est maintenant sous une polychimiothérapie à base de gemcitabine et docetaxel. Lorsqu'un léiomyosarcome est découvert histologiquement sur une pièce d'hystérectomie, aucun traitement adjuvant n'est à prévoir et notamment il n'est pas nécessaire de réintervenir pour un stading ganglionnaire, le léiomyosarcome étant peu lymphophile. Toutefois, il est préférable de réaliser un bilan d'extension afin de s'assurer qu'il n'existe pas de métastases pulmonaires et abdominales (radiographie pulmonaire et scanner abdomino-pelvien). Selon le risque histologique d'effraction utérine une radiothérapie postopératoire peut être proposée.

## Conclusion

Le léiomyosarcome est un cancer rare, mais dont le pronostic reste sombre. Le diagnostic pré-opératoire n'est que rarement fait; Le léiomyosarcome se présente le plus souvent sous la forme d'un myome banal ou en nécrobiose. Le principal facteur pronostique est l'activité mitotique. Le traitement est dominé par la chirurgie. La radiothérapie ne permet que de diminuer les récidives locales sans modifier la survie et la chimiothérapie n'a pas fait la preuve de son efficacité.
